# Impact of Nutrition Counseling in Head and Neck Cancer Sufferers Undergoing Antineoplastic Therapy: A Randomized Controlled Pilot Study

**DOI:** 10.3390/curroncol29100546

**Published:** 2022-09-26

**Authors:** Wangshu Dai, Shu-An Wang, Kongcheng Wang, Chen Chen, Juan Wang, Xiaotian Chen, Jing Yan

**Affiliations:** 1 Department of Geriatric Medcine, Nanjing Drum Tower Hospital, The Affiliated Hospital of Nanjing University Medical School, Nanjing 210008, China; 2 Department of Clinic Nutrition, Nanjing Drum Tower Hospital, The Affiliated Hospital of Nanjing University Medical School, Nanjing 210008, China; 3 The Comprehensive Cancer Center of Drum Tower Hospital, Medical School of Nanjing University, Clinical Cancer Institute of Nanjing University, Nanjing 210008, China

**Keywords:** head and neck cancer, nutrition counseling, weight loss, anxiety, depression

## Abstract

Head and neck cancer (HNC) sufferers usually encounter arduous nutritional problems when they are receiving antineoplastic therapy. Consequently, the presence of anxiety and depression is commonly observed in this population. This study aimed to explore the physical and psychological influence of nutritional counseling in patients with HNC. Patients receiving concurrent chemo-radiotherapy were randomly assigned to the nutritional counseling group (*n* = 32, 52.45%) and the control group (*n* = 29, 47.54%) according to their treatment patterns. In the nutritional counseling group, registered dietitians provided face-to-face counseling during the antineoplastic treatment course at least every two weeks. Nutrient intake amount, relevant nutritional indexes, quality of life, and the degree of anxiety and depression were compared between the two groups. We observed a decrease in the calorie and protein intake amount in both groups, while the decrease in the control group is even worse. The weight loss is more obvious in the control group. The HADS scores in the intervention group were significantly lower than that in the control group (*p* < 0.05). The Karnofsky Performance Status (KPS) scores in the intervention group were significantly higher than that in the control group (*p* < 0.05). The level of serum total protein, serum albumin, transferrin, and the thickness of the triceps skin fold decreased less in the intervention group (*p* < 0.05). Our findings suggest that nutrition counseling is essential for the maintenance of calorie and protein intake in HNC suffers, which contributes to an improvement in the physical and psychological states. The impacts observed in this pilot study warrant further exploration in a larger prospective trial.

## 1. Introduction

Head and neck cancer (HNC) is the eighth most common cancer in the world [1]. The development of this disease may be associated with the infection of oncogenic viruses such as human papillomavirus (HPV) and Epstein-Barr virus (EBV). Globally, the incidence of HNC has increased during the decades partly due to an increase in oncogenic virus infection. Over 900,000 individuals are diagnosed with HNC per year [2]. Tumor related hyper-metabolic effects and side effects of anti-tumor therapy both result in cancer patients’ malnutrition [3,4]. The prevalence rate of malnutrition is approximately 30–80% among cancer sufferers. HNC sufferers are one of the most susceptible groups in terms of malnutrition before, during, and after antineoplastic treatment [5,6]. Concurrent chemo-radiotherapy (CCRT) is the standard curative treatment modality for this population [2]. The specific anatomical location of HNC brings side effects from antineoplastic therapy such as taste changes, oral cavity dryness, mucositis, nausea and vomiting, and poor appetite [7,8]. These disorders impair the individual’s ability to eat and impede nutrition intake [9].

It was reported that unwilling weight loss in HNC suffers during and after the treatment course relate to poorer prognosis [10,11,12]. Malnutrition in this population represents increased morbidity and mortality, and worsens health-related quality of life [13,14]. Facing the diagnosis of cancer negatively influences the mental state of patients, which takes a turn for the worse with repulsive symptoms and side effects of radical CCRT [15]. The incidence of suicide in HNC suffers was more than three times higher than that in other types of cancer [16].

Both the biopsy process and the treatment of HNC suffers, which includes radiation, surgery, and chemotherapy, have adverse influences on patients’ health related quality of life [17]. Distortions of voice, taste, chewing, swallowing, hearing, and breathing may produce psychological burden for decades after the end of the treatment course [18]. Critical weight loss (5%) during treatment was observed to affect 66% of patients with HNC [19]. Nutritional recommendations for HNC sufferers should aim for 1.2 g/kg protein daily. Daily energy intake should be more than or equal to 125 kJ/kg (30 kcal/kg) and it is crucial to monitor body weight and nutritional status periodically and adjust nutrient intake as required [3].

Intervention to add nutrition counseling to the treatment course of this population is necessary. In order to maintain adequate nutritional intake for HNC sufferers, nutritional compliance may arouse patients’ attention to adequate dietary intake and mobilize patients to partly participate in cancer treatment [7]. This trial was designed to evaluate the efficacy of nutrition counseling in HNC patients. Our study aimed to conduct a preliminary evaluation of whether nutrition counseling is an effective treatment for the management of HNC patients.

## 2. Materials and Methods

### 2.1. Patients

This was a prospective study involving HNC sufferers receiving radical CCRT from June 2018 to December 2019 at the Comprehensive Cancer Centre of Drum Tower Hospital, Clinical Cancer Institute of Nanjing University. In all cases, written informed consent was obtained. A multidisciplinary team participated in the diagnosis of HNC followed by the NCCN guidelines. The study protocol was approved by the ethics committee of Nanjing Drum Tower Hospital (2018-072-01). This study was registered at chictr.org.cn (ChiCTR1800016070). Patients were included in our study referring to the following criteria: (i) aged between 18 and 75 years; (ii) pathologically diagnosed with HNC (with clinical stage of III or IV) and were prescribed CCRT; (iii) grade of B or C on the patient-generated subjective global assessment (PG-SGA); The exclusion criteria were as follows: (i) participated in other clinical research, (ii) with evidence of cognitive dysfunction that would limit their ability to communicate, (iii) had an allergic history of enteral nutrition supplements, (iv) with a life expectancy less than 3 months, (v) along with other types of cancer (vi) had a history of spirit medicine intake within 45 days. (vii) Diabetic patients with poor blood glucose control, (viii) patients with intestinal absorption diseases. Patients enrolled were randomly assigned to the nutritional counseling group and the control group according to their treatment patterns.

### 2.2. Definitive Concurrent Chemo-Radiotherapy

All patients received intensity modulated radiation therapy (IMRT) or volumetric modulated arc therapy (VMAT) at a conventionally fractionated regimen of 200 cGy per day for 5 consecutive days weekly, with a total prescribed radiation dose of 7000–7400 cGy. The initial target volume included the tumor area and regional drainage area of the lymph node. After receiving irradiation of 4600–5000 cGy, the radiation field was limited to only the tumor area and the regional lymph nodes. Nedaplatin (30 mg/m^2^ every week) was administered concurrently with radiotherapy referring to the treatment protocols at our cancer center.

### 2.3. Nutrition Counseling

A registered dietician provided face-to-face nutritional counseling to each patient in the counseling group at least once every 2 weeks during the CCRT course. Each patient receives individualized dietary counseling to achieve enough estimated calorie and protein requirements. Patients who failed to meet 80% goals of calorie and protein requirements were provided with an oral nutrition supplement (ONS). Patients who were unable to keep their body weight for two consecutive evaluation cycles despite the use of ONS were required to start enteral or parenteral nutrition support. Details were described in the Supplemental Document. In the control group, clinicians designed nutrition treatment plans according to clinical experience.

### 2.4. Follow Up

Calorie and protein intake, body weight, along with physical and mental state were evaluated as follow-up indicators. The actual calorie and protein intake was evaluated by the method of 24 h dietary investigation. Physical and mental state assessment was performed referring to Karnofsky Performance Status (KPS) score and Hospital Anxiety and Depression Scale (HADS), respectively. The patients were followed up at four time spots: baseline, two weeks, four weeks, and six weeks after the initiation of CCRT. The evaluations were performed a day before and after the cut-off time point. In addition, Measurement of thickness of triceps skin fold (TSF) and laboratory tests including serum total protein, serum albumin, pre-albumin, and transferrin assays were performed before and after the treatment course.

### 2.5. Statistical Analyses

The analysis of patient characteristics was based on the intention-to-treat (ITT) population. The characteristic data was presented as median and range. Other data were presented as mean and standard deviation. Students’ t tests were used for continuous variables and the chi-square test (and Fisher’s exact) for categorical variables. All analyses were performed using SPSS software (version 18.0, IBM Corp., Armonk, NY, USA). A *p* value less than 0.05 was considered statistically significant.

## 3. Results

### 3.1. Patient Demographics and Clinical Characteristics

**Figure 1:**
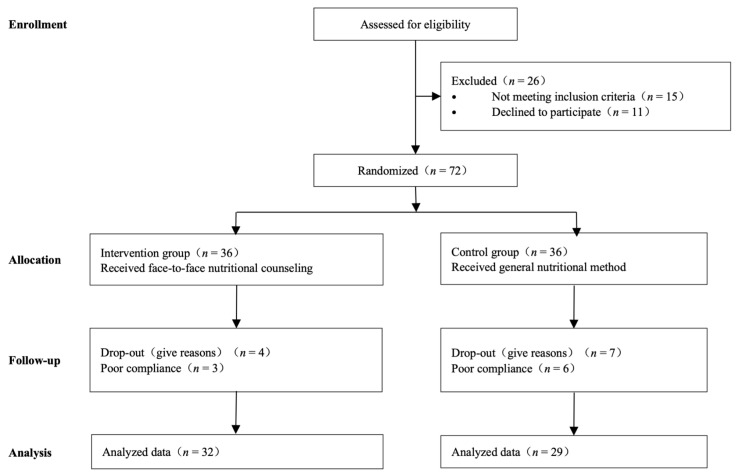
Flow of participants, participant screening, randomization, and follow-up.

A total of 61 patients were enrolled and evaluated in our trial (Figure 1). These patients were randomly allocated to the nutrition counseling group and control group. In the nutrition counseling group, two patients were in stage III, and 30 patients were in stage IV. In the control group, two patients were in stage III, and 27 patients were in stage IV. Patient characteristic files at baseline are summarized in Table 1. Generally, 81.97% of subjects were men, and the average age was 51.67 years. Accordingly, 32 (52.46%), and 29 (47.54%) subjects were allocated to the nutritional counseling and control groups, respectively. The majority of the subjects (83.61%) had nasopharyngeal cancer. No statistical differences between the two groups were observed regarding sex, age, height, weight, educational level, and tumor types. A total of two patients enrolled in our study have a history of diabetes, and the blood glucose of these two patients is well controlled. None of the enrolled subjects suffered intestinal absorption diseases such as inflammatory bowel disease, dyspepsia, diarrhea, etc. All patients enrolled in this study suffered from mucositis of different degrees. In the intervention group, mucositis incidence (RTOG standards) was 7 in level I (21.88%), 20 in level II (62.50%), 5 in level III (15.62%), and 0 in level IV (0.00%); In the control group, mucositis incidence was 3 in level I (10.35%), 15 in level II (51.72%), 9 in level III (31.03%), and 2 in level IV (6.90%).

### 3.2. Nutrient Intake

**Figure 2:**
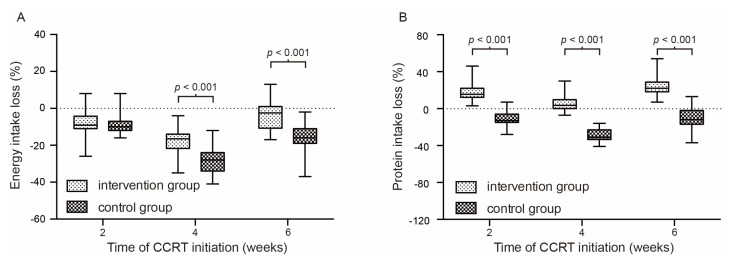
Percentage of energy intake change (**A**) and protein intake change (**B**) at different time nodes of concurrent chemo-radiotherapy.

The calorie intake in the intervention group is significantly better than those in the control group since the fourth week (*p* < 0.05). The maintenance of protein intake is significantly better than those in the control group. The ratio of energy nitrogen ratio was kept at a lower level in the intervention group throughout the treatment course. Details are summarized in Table 2. Figure 2 shows the nutrient intake change at different time nodes of concurrent chemo-radiotherapy.

### 3.3. Physical State Assessment

**Figure 3:**
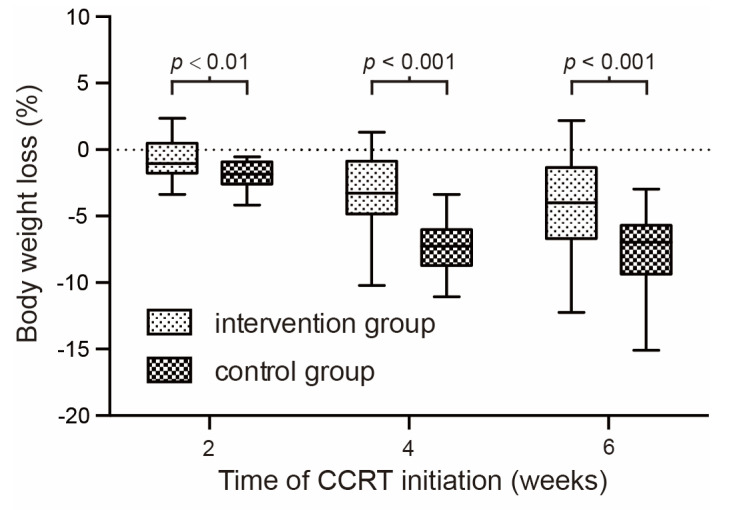
Percentage of body weight change at different time nodes of concurrent chemo-radiotherapy.

Weight loss is even more serious in the control group. Table 3 and Table 4 show the body weight change and KPS score in the second month, the fourth month, and the sixth month of the CCRT course. A significant difference was observed since the fourth week. The KPS score of quality of life in the counseling group is significantly better than that in the control group since the fourth week. Figure 3 shows the body weight change at different time nodes of concurrent chemo-radiotherapy.

### 3.4. Mental State Assessment

The results of the HADS-A and HADS-D questionnaires are summarized in Table 5. High rates of anxiety and depression were found after CCRT therapy. Patients got the highest scores in anxiety and depression in the fourth week. The anxiety and depression of patients had eased in the sixth week. Nutrition counseling could ameliorate the emotional disturbances, and statistically significant differences were observed since the second week.

### 3.5. Results of Nutritional Testing

To evaluate the effectiveness of nutritional counseling, blood biochemical tests including total protein, albumin, transferrin, and pre-albumin were performed and the thickness of triceps skin fold (TSF) of patients was measured. Baseline levels and deltas of nutritional indexes are recorded in Table 6. The decline of albumin, transferrin, and pre-albumin was significantly alleviated in the intervention group compared with the control group. The level of total protein in the intervention group shows no sign of waning.

## 4. Discussion

The study explored the influence of nutritional counseling on HNC sufferers receiving CCRT. Current studies reported that HNC sufferers had a significantly better prognosis if they received nutritional counseling during the CCRT course [6]. Nutritional counseling helps to maintain nutrient intake amount and makes a great contribution to the prevention of body weight loss, which improved tolerability of the loathsome treatment and treatment-related side effects to some extent [20]. All the same, HNC sufferers’ nutritional status is changing and deteriorating throughout the CCRT course, which results in patients’ malnutrition [21]. As previous studies described, a large proportion of HNC sufferers with normal nutritional status at baseline developed to be malnourished after the CCRT course. CCRT resulted in an average body weight loss of 5 to 7.4% in HNC patients [5]. It is reported that nutritional counseling positively increased nutrient intake amount, enhanced the quality of life, and alleviated treatment-related toxicities in HNC sufferers undergoing CCRT [22]. In our study, a decrease in the calorie intake amount and body weight were observed in both groups, while the decrease in the control group is even worse. As nutritional counseling brings the application of nutritional support, weight loss is alleviated in the intervention group. In addition, we observed that the maintenance of protein intake is significantly better than those in the control group; as a consequence, the energy nitrogen ratio was kept at a lower level throughout the treatment course which is beneficial to cancer patients.

Current studies showed that the psychological and nutritional statuses of HNC patients were interconnected [23]. HNC sufferers have a higher incidence of psychological disorders and malnutrition than other patients with cancer [15]. Patients live in different social and economic environments, and as a consequence, the level of psychological distress is diversified. We used the HADS questionnaire to evaluate the mental state of HNC sufferers receiving CCRT. In our study, the prevalence of anxiety and depression peaked in the fourth week after treatment. Nutrition counseling could ameliorate the emotional disturbances, and statistically significant differences were observed since the second week.

Blood biochemical tests including total protein, albumin, transferrin, and pre-albumin were performed and thicknesses of triceps skin fold (TSF) of patients were measured to evaluate the nutritional status of HNC patients. In our study, we observed a decline in albumin, transferrin, and pre-albumin level despite nutritional counseling was offered to the HNC sufferers, indicating the limited efficiency of nutritional counseling and support. Westin et al. performed a study on the interaction of the mental and nutritional status of HNC sufferers 30 years ago [24]. They found that depression increased the incidence of malnutrition one year after the end of treatment. Those studies suggest that psychological support is recommended to achieve a greater efficacy of nutritional counseling.

This study has some limitations. Insufficient sample size limits the accuracy and authenticity of the results. Treatment completion data for some patients are missing due to force majeure factors. The lack of follow-up data contributes to the lack of survival profiles. These limitations impair the comprehensive interpretation of the potency of nutrition counseling. Validation by a larger randomized trial and a long-term follow up is necessary in future studies.

## 5. Conclusions

HNC sufferers undergoing CCRT therapy usually encounter complex nutritional problems owing to the specific anatomic locations of cancer. Both the disease itself and the treatment do harm to organs that are vital for the maintenance of swallowing function, which further impairs the sufferer’s ability to maintain adequate nutrient intake. Our study showed nutrition counseling is beneficial to maintain a patient’s nutrition intake, which positively impacts the physical and psychological states of HNC suffers. Further exploration in a larger prospective trial is needed.

## Figures and Tables

**Table 1:** Basic characteristics of the patients (Mean ± SD); Abbreviations: HADS-A, Hospital Anxiety and Depression Scale- Anxiety; HADS-D, Hospital Anxiety and Depression Scale- Depression; KPS, Karnofsky Performance Status; ONS, Oral Nutrient Supplements.

Category	Intervention Group (*n* = 32)	Control Group (*n* = 29)	*p* Value
Age	51.7 ± 12.9	56.0 ± 8.3	0.12
Sex			
Male	27	23	0.74
Female	5	6	
Height (m)			
Male	1.68 ± 0.07	1.66 ± 0.07	0.49
Female	1.59 ± 0.07	1.55 ± 0.05	0.19
Weight (kg)			
Male	69.6 ± 11.6	66.8 ± 9.8	0.45
Female	65.1 ± 10.3	58.3 ± 6.4	0.22
Calorie intake (kcal/d)	1642.1 ± 183.0	1614.1 ± 201.5	0.57
Protein intake (g/d)	54.3 ± 6.9	52.8 ± 6.8	0.40
Energy nitrogen ratio	190.2 ± 16.9	191.4 ± 8.3	0.73
HADS-A score	5.8 ± 1.3	5.3 ± 1.8	0.31
HADS-D score	4.3 ± 1.7	4.3 ± 1.2	0.94
KPS score	91.6 ± 7.7	91.0 ± 6.7	0.78
Educational level			
High school graduate	9	6	0.56
Less than high school	23	23	
Tumor site			
Nasopharynx	27	24	1.00
Hypo-pharynx	1	2	
Tonsil	1	0	
Cervical lymph node	0	1	
Tongue	0	1	
Larynx	2	1	
Oropharynx	1	0	
Nutrition support method			
Dietary	9	7	0.732
ONS	21	20	
Tube feeding	2	2	

**Table 2:** Comparison of calories and proteins intake between the two groups (Mean ± SD).

Category	Second Week	Fourth Week	Sixth Week	F	*p* Value
Calorie difference					
Intervention group (*n* = 32)	−136.2 ± 108.5	−308.2 ± 130.1	−72.0 ± 122.1	21.32	0.00
Control group (*n* = 29)	−150.7 ± 77.6	−466.8 ± 126.0	−251.0 ± 113.0		
Protein difference					
Intervention group (*n* = 32)	9.1 ± 4.4	2.3 ± 3.9	12.6 ± 4.7	223.71	0.00
Control group (*n* = 29)	−5.9 ± 4.5	−15.0 ± 5.1	−6.2 ± 6.7		
Energy nitrogen ratio					
Intervention group (*n* = 32)	149.3 ± 13.3	147.7 ± 10.6	147.5 ± 11.8	190.56	0.00
Control group (*n* = 29)	195.9 ± 12.6	190.4 ± 19.0	184.0 ± 25.0		

**Table 3:** Comparison of weight between the two groups (Mean ± SD).

Category	Second Week	Fourth Week	Sixth Week	F	*p* Value
Weight					
Intervention group (*n* = 32)	68.3 ± 10.8	66.7 ± 10.3	66.1 ± 10.3	4.86	0.03
Control group (*n* = 29)	63.9 ± 9.8	60.2 ± 9.5	60.0 ± 9.0		

**Table 4:** Comparison of KPS between the two groups (Mean ± SD); Abbreviation: KPS, Karnofsky Performance Status.

Category	Second Week	Fourth Week	Sixth Week	F	*p* Value
KPS score					
Intervention group (*n* = 32)	81.3 ± 6.6	70.6 ± 6.7	65.6 ± 5.0	8.16	0.01
Control group (*n* = 29)	78.6 ± 7.9	66.6 ± 6.7	62.1 ± 4.9		

**Table 5:** Comparison of HADS-A and HADS-D between the two groups (Mean ± SD); Abbreviations: HADS-A, Hospital Anxiety and Depression Scale- Anxiety; HADS-D, Hospital Anxiety and Depression Scale- Depression.

Category	Second Week	Fourth Week	Sixth Week	F	*p* Value
HADS-A score					
Intervention group (*n* = 32)	4.6 ± 1.5	5.5 ± 1.8	3.6 ± 1.0	8.77	0.00
Control group (*n* = 29)	5.2 ± 1.5	6.3 ± 1.1	4.5 ± 1.0		
HADS-D score					
Intervention group (*n* = 32)	3.7 ± 0.8	4.8 ± 1.2	3.8 ± 1.2	6.97	0.01
Control group (*n* = 29)	3.8 ± 1.0	5.5 ± 1.2	4.8 ± 1.2		

**Table 6:** Comparison of nutritional indexes between the two groups (Mean ± SD).

Category		Intervention Group (*n* = 32)	Control Group (*n* = 29)	t	*p* Value
TP (g/L)	Baseline level	70.4 ± 11.7	73.6 ± 5.9	−1.32	0.19
	Deltas against the baseline	0.7 ± 13.0	−5.5 ± 6.9	2.31	0.02
ALB (g/L)	Baseline level	42.8 ± 3.1	42.0 ± 2.9	1.03	0.31
	Deltas against the baseline	−1.4 ± 3.4	−3.1 ± 2.9	2.06	0.04
TF (g/L)	Baseline level	2.0 ± 0.4	2.1 ± 0.3	−0.44	0.67
	Deltas against the baseline	−0.1 ± 0.5	−0.3 ± 0.4	2.19	0.03
PA (mg/L)	Baseline level	251.8 ± 59.2	232.0 ± 68.3	1.20	0.24
	Deltas against the baseline	−26.4 ± 71.3	−56.7 ± 62.5	1.75	0.08
TSF (mm)	Baseline level	136.3 ± 15.7	133.1 ± 18.8	−0.90	0.38
	Deltas against the baseline	−6.0 ± 10.5	−9.8 ± 12.4	2.01	0.04

## Data Availability

The raw data required to reproduce these findings cannot beshared at this time as the data also forms part of an ongoing study.

## Supplementary Materials

The following supporting information can be downloaded at: https://www.mdpi.com/article/10.3390/curroncol29100546/s1, Supplemental Document: Nutrition intervention protocol.
