# Supplementary material for: Impact of Nutrition Counseling in Head and Neck Cancer Sufferers Undergoing Antineoplastic Therapy: A Randomized Controlled Pilot Study
Source: Curr Oncol. 2022 Sep 26;29(10):6947–55. doi: 10.3390/curroncol29100546 (PMC9600229; doi:10.3390/curroncol29100546)
Supplement: Supplementary file 1 [file curroncol-29-00546-s001.zip › curroncol-1876854-supplementary.pdf]

Current Oncology

Nutrition intervention protocol

Nutritional support programmes were established for subjects enrolled in both intervention and control group according to the principles recommended by "ESPEN guidelines on nutrition in cancer patients "during the concurrent chemo-radiotherapy course. Energy intake were kept between 25 and 30 kcal/kg/day; Protein intake were kept between 1.0 and 2.0 g/kg/day; Liquid volume were kept between 30 and 40 mL/kg/day. Among the total energy sources, carbohydrate accounts for 30% ~ 50%, protein accounts for 15% ~ 30%, and fat accounts for 25% ~ 40%.

In the intervention group, patient-generated subjective global assessment (PG-SGA), dietary survey, hematological examination, treatment response assessment to each patient were performed by a registered dietician at least once every 2 weeks. The dietician provided face-to-face nutritional counseling and carried out whole process nutrition management for each patient. If the PG-SGA score ranged between 0 to 3 points, diet guidance was conducted (Dietary types include ordinary dietsoft diet, semiliquid food and liquid food.). If the PG-SGA score is greater than 4 points, and the actual diet intake fails to meet the target of 80% calories and protein requirements, the patient shall be given oral nutrition supplement. After given oral nutrition supplement, patients who could not maintain their weight for two consecutive assessment cycles were asked to start enteral or parenteral nutrition support. Nutritional support and assessment were adjusted over time until discharged for hospital.

In the control group, whether to give artificial nutritional support is determined by clinical oncologists according to the general diet and hematological examination results of the patients but not nutritional assessment and dietary survey.
